# Enhanced Transdermal Delivery of Pranoprofen Using a Thermo-Reversible Hydrogel Loaded with Lipid Nanocarriers for the Treatment of Local Inflammation

**DOI:** 10.3390/ph15010022

**Published:** 2021-12-24

**Authors:** María Rincón, Marcelle Silva-Abreu, Lupe Carolina Espinoza, Lilian Sosa, Ana Cristina Calpena, María J. Rodríguez-Lagunas, Helena Colom

**Affiliations:** 1 Department of Materials Science and Physical Chemistry, Faculty of Chemistry, University of Barcelona, C. Martí i Franquès 1–11, 08028 Barcelona, Spain; m.rincon@ub.edu; 2 Department of Pharmacy, Pharmaceutical Technology and Physical Chemistry, Faculty of Pharmacy and Food Sciences, University of Barcelona, Av. Joan XXIII 27–31, 08028 Barcelona, Spain; anacalpena@ub.edu (A.C.C.); helena.colom@ub.edu (H.C.); 3 Institut de Nanociència i Nanotecnologia IN2UB, University of Barcelona, 08028 Barcelona, Spain; 4 Departamento de Química, Universidad Técnica Particular de Loja, Loja 1101608, Ecuador; lcespinoza@utpl.edu.ec; 5 Faculty of Chemical Sciences and Pharmacy, National Autonomous University of Honduras (UNAH), Tegucigalpa 11101, Honduras; liliansosa2012@gmail.com; 6 Department of Biochemistry and Physiology, Faculty of Pharmacy and Food Sciences, University of Barcelona, 08028 Barcelona, Spain; mjrodriguez@ub.edu; 7 Nutrition and Food Safety Research Institute (INSA-UB), 08921 Santa Coloma de Gramenet, Spain

**Keywords:** pranoprofen, thermo-reversible hydrogel, nanostructured lipid carriers, transdermal delivery, inflammation

## Abstract

A biocompatible topical thermo-reversible hydrogel containing Pranoprofen (PF)-loaded nanostructured lipid carriers (NLCs) was studied as an innovative strategy for the topical treatment of skin inflammatory diseases. The PF-NLCs-F127 hydrogel was characterized physiochemically and short-time stability tests were carried out over 60 days. In vitro release and ex vivo human skin permeation studies were carried out in Franz diffusion cells. In addition, a cytotoxicity assay was studied using the HaCat cell line and in vivo tolerance study was performed in humans by evaluating the biomechanical properties. The anti-inflammatory effect of the PF-NLCs-F127 was evaluated in adult male Sprague Daw-ley^®^ rats using a model of inflammation induced by the topical application of xylol for 1 h. The developed PF-NLCs-F127 exhibited a heterogeneous structure with spherical PF-NLCs in the hydrogel. Furthermore, a thermo-reversible behaviour was determined with a gelling temperature of 32.5 °C, being close to human cutaneous temperature and thus favouring the retention of PF. Furthermore, in the ex vivo study, the amount of PF retained and detected in human skin was high and no systemic effects were observed. The hydrogel was found to be non-cytotoxic, showing cell viability of around 95%. The PF-NLCs-F127 is shown to be well tolerated and no signs of irritancy or alterations of the skin’s biophysical properties were detected. The topical application of PF-NLCs-F127 hydrogel was shown to be efficient in an inflammatory animal model, preventing the loss of stratum corneum and reducing the presence of leukocyte infiltration. The results from this study confirm that the developed hydrogel is a suitable drug delivery carrier for the transdermal delivery of PF, improving its dermal retention, opening the possibility of using it as a promising candidate and safer alternative to topical treatment for local skin inflammation and indicating that it could be useful in the clinical environment.

## 1. Introduction

Inflammation is a protective response of the body’s tissues to irritation or injury. It can be classified as an acute or chronic process. Its cardinal signs are redness, fever, swelling, and pain, accompanied by functional impotence. The most common skin inflammatory diseases are atopic dermatitis, rosacea, vitiligo, psoriasis, eczema, and panniculitis [1].

**Figure 1:**
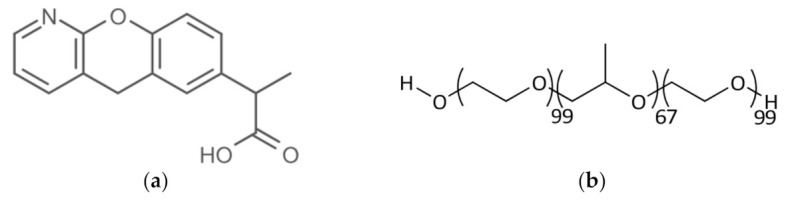
(**a**) Pranoprofen chemical structure [2]; (**b**) Pluronic F127^®^ chemical structure [15].

Pranoprofen (PF), or 2-(5H-chromeno[2,3-b]pyridin-7-yl)propanoic acid [2] (Figure 1a), is a potent non-steroidal anti-inflammatory drug (NSAID) used to treat pain, fever, and inflammation, as well as it being a drug alternative for the treatment of osteoarthritis, rheumatoid arthritis, and ocular therapy [3,4,5]. PF eliminates the inflammation by inhibiting the expression of the cyclooxygenase (COX) enzyme and consequently reduces the prostaglandin synthesis from arachidonic acid [6]. Nevertheless, it presents low bioavailability due to its poor solubility in water, has a short plasmatic half-life and it is unstable in aqueous solutions, particularly when exposed to light [7,8].

Nowadays, the drug delivery systems are an alternative way to improve the drug’s bioavailability, delivery to a specific place, its controlled release, as well as providing protection of the incorporated drug from the external environment. Furthermore, these systems are transporters of protein, macromolecules, liposoluble and hydrophilic drugs. Moreover, they reduce the side effects, present efficient penetration reducing drug toxicities and improve the therapeutic efficacy of the drugs [9]. Nanostructured lipid carriers (NLCs) are a delivery system composed of a mixture of liquid lipid (oil) and solid lipid that generates an imperfect crystalline matrix and this allows the solubilization and incorporation of the drug into the lipid mixture. NLCs are very useful for skin application due to their characteristics of film formation, skin hydration, controlled occlusion, enhanced skin bioavailability and physical stability [10].

Hydrophilic gels (hydrogels) are cross-linked three-dimensional networks that can serve as efficient drug depots to supply local drug delivery. They react to endogenous or exogenous triggers, that can swell and retain a large amount of water without dissolution [11]. By modifying the hydrogels’ density, the rate at which an entrapped drug is released can be modified, and this enables the delivery of the drug over a concrete period [12]. They are defined by the European Pharmacopoeia as preparations, the bases of which usually consist of water, glycerol, or propylene glycol gelled with suitable gelling agents, such as poloxamers, starch, cellulose derivatives, carbomers, and magnesium–aluminium silicates [13].

The term Pluronic^®^ (also known as Poloxamer) identifies a generic block copolymer made up of two units of poly(ethylene glycol) (PEG), separated by a central poly(propylene oxide) unit (PPO). One of the different varieties of Pluronic^®^ is the F127 (Figure 1b), which is commercially available due to its physiological properties of low toxicity and high biocompatibility [14]. Furthermore, it displays a double behaviour. Depending on the temperature and concentration used, it could be a Newtonian or viscoelastic solution (solution-gel transition) [15,16]. Its advantage giving properties are that it promotes and improves the drug permeation through the skin and mucosa [17,18].

From a biopharmaceutical viewpoint, the skin is recognized as an interesting route for drug delivery. Overall, small molecules can penetrate the stratum corneum, which is the major barrier and the external layer of the skin [19]. The percutaneous application of NSAIDs offers the advantages of delivering the drug at a sustained level over an extended time period and the avoidance of the hepatic first-pass effect. Moreover, it acts mainly at the joints and related regions, and the drug can be concentrated at the inflammation site [20].

The purpose of this study was to develop and characterize a new topical Pluronic^®^ F127 hydrogel loaded with NLCs of PF (PF-NLCs-F127 hydrogel) in order to modulate the drug release and promote its retention in human skin in the treatment of local inflammations.

## 2. Results

### 2.1. Composition and Physicochemical Characterization of the PF-NLCs

Firstly, the selected PF-NLCs suspension was described, optimized, and developed [21]. The final composition of the PF-NLCs is shown in Table 1.

PF (cPF); Tween^®^ 80 (cTW); lipid phase (L) (5% of total formulation) which is composed of LAS: Castor oil (75:25) as liquid lipid (LL), and Precirol^®^ ATO 5 as solid lipid (SL), (cSL/L).

Describing it briefly, the developed PF-NLCs exhibits a mean particle size (Z-Ave) of around 248.40 nm, a negative surface charge with Zeta potential (ZP) values around −10.70 mV, polydispersity index (PI) values lower than 0.3, which indicates a monomodal distribution. The Encapsulation efficiency percentage (EE%) shows values of around 100 % (Table 1).

### 2.2. Physicochemical Characterization of the PF-NLCs-F127 Hydrogel

#### 2.2.1. Appearance, pH Evaluation

The PF-NLCs-F127 hydrogel exhibited a whitish-translucent colour, smooth, homogeneous appearance, and some limited flowability when the vial was inverted. This formulation showed a pH of 6.5, which is biocompatible with the skin [22]. After one month of storage at room temperature 25 ± 2 °C, and 60 ± 5% of relative humidity (RH), no changes were detected.

#### 2.2.2. Morphological Studies

**Figure 2:**
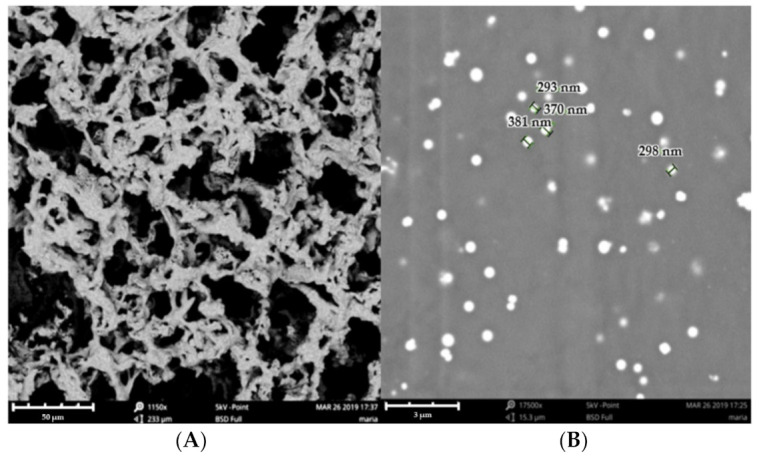
(**A**) Image of PF-NLCs-F127 hydrogel by SEM (structure); (**B**) Image of PF-NLCs-F127 hydrogel by SEM (size).

Scanning electron microscopy (SEM) was used to evaluate the morphology of the hydrogel. The particles seemed to be homogeneously dispersed in the hydrogel (Figure 2A), which exhibited a heterogeneous structure with the formation of interconnected capillary channels in the form of holes similar to those of a porous sponge.

Figure 2B shows particles of spherical shape and size that ranged between 293 ± 2.34 nm and 381 ± 1.23 nm.

#### 2.2.3. Gelling Temperature

The experiment was carried out to determine the temperature of the hydrogel at which a change in the fluidity of the formula was observed. The temperature transition of the formulation to gelling was at 32.5 °C. The data were obtained from the mean of three experiments (*n* = 3).

#### 2.2.4. Swelling, Degradation and Porosity Studies

**Figure 3:**
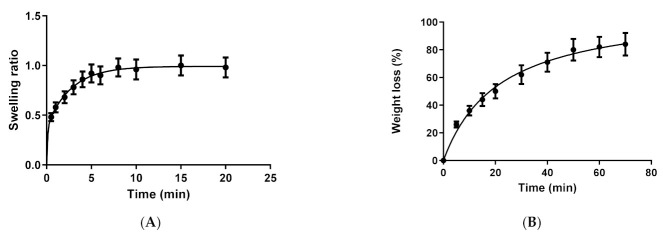
(**A**) Swelling ratio of PF-NLCs-F127 upon immersion in PBS pH = 5.5; (**B**) percentage of weight loss degradation of PF-NLCs-F127 in PBS pH = 5.5.

The swelling experiment was carried out in PF-NLCs-F127 hydrogel so as to evaluate its capacity to include solvent beneath the matrix. Dehydrated hydrogel was immersed in phosphate buffer saline (PBS) (pH 5.5), and its weight was recorded at certain time intervals. The swelling process of PF-NLCs-F127 followed a second-order kinetic model, which was represented by the kinetic constants K1 = 0.26 min^−1^ and K2 = 9.128 min^−1^. After 7 min, practically 100% swelling was achieved (Figure 3A).

Additionally, the degradation experiments were carried out by immersing hydrogels in PBS (pH 5.5) and recording their weight at certain intervals. The degradation process of the formula was completed in 1 h and it followed a hyperbola model with a kinetic constant (Kd) of 21.64 min^−1^. Figure 3B shows the degradation profile, after 1 h 100% of the formulation was degradated. The porosity percentage was 95.76 ± 0.1% for the formulation.

#### 2.2.5. Rheological Behaviour

**Figure 4:**
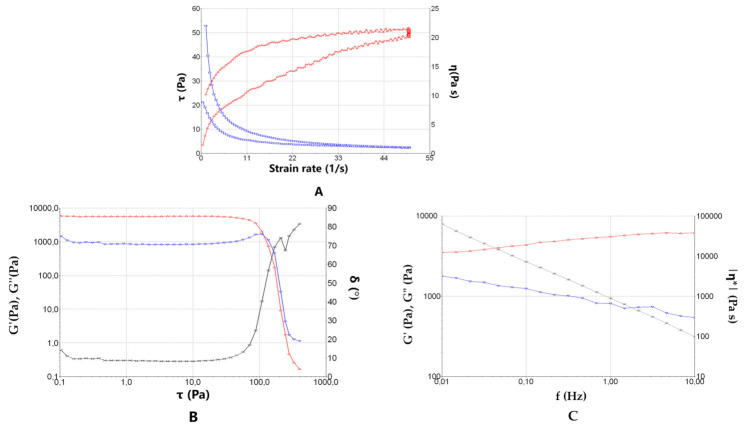
(**A**) Rheogram of PF-NLCs-F127 hydrogel at 25 °C. The flow curve represents shear stress (τ in Pa) (left axis) or viscosity (η in Pa·s) (right axis) as a function of the shear rate (γ in s^−1^). (**B**) Oscillatory study applied to PF-NLCs-F127 hydrogel at 32 °C. Variation of elastic modulus G′ (**in red**), viscous modulus G′′ (**in blue**) and the phase angle δ as a function of shear stress τ. (Frequency constant 1 Hz) (**C**) Oscillatory study applied to PF-NLCs-F127 hydrogel, at 32 °C. Variation of elastic modulus G′ (**in red**), viscous modulus G′′ (**in blue**) and the phase angle δ as a function of shear stress π (τ constant 1 Pa). The data were obtained from the mean of three experiments (*n* = 3).

The rheological behaviour plays an integral role in evaluating the overall efficiency of topical/transdermal formulation. The shear rate increased at the beginning and afterwards decreased, throughout testing, shear stress (τ) and viscosity (η) were measured (Figure 4A).

The flow curve of the hydrogel indicated no thixotropic behaviour in the system, as the rheogram did not display any hysteresis loop. The mathematical model that best fitted the test data in compliance with the highest value of the correlation coefficient of regression (r) was Cross, which indicates a pseudoplastic behaviour, with values of (r) of 0.9984 and 0.9981 for ascending and descending sections of the flow curve, respectively. Beforehand, the viscosity measurements from the constant share rate period of 50 s^–1^ at 25 °C was 1.004 ± 0.018 Pa·s, showing good repeatability of the rheological results between samples. At 32 °C, the flow and viscosity curve plots were excessively irregular since the hydrogel was too viscous at this temperature and did not flow adequately under the experimental conditions. Although the gel did not flow easily at 32 °C, it exhibited interesting viscoelastic properties. Figure 4B shows the result of the shear sweep test of the oscillatory study applied to PF-NLCs-F127, at 32 °C, and a frequency constant of 1 Hz. This allows the establishment of the critical limit of effort (moment in which there is a great variation of the modules G′ and G′′), in 30 Pa. In the zone of linear viscoelasticity, the elastic modulus (G′ red colour) predominates over the viscous modulus (G′′ blue colour). Figure 4C shows the result of the scan frequency test of the oscillatory study applied to the PF-NLCs-F127, at 32 °C at a constant effort of 1 Pa, which is within the zone of linear viscosity. The prevalence of the elastic part over the viscous part is confirmed (given that G′ is always greater than G′′) for this hydrogel.

#### 2.2.6. Extensibility (Spreadability)

**Figure 5:**
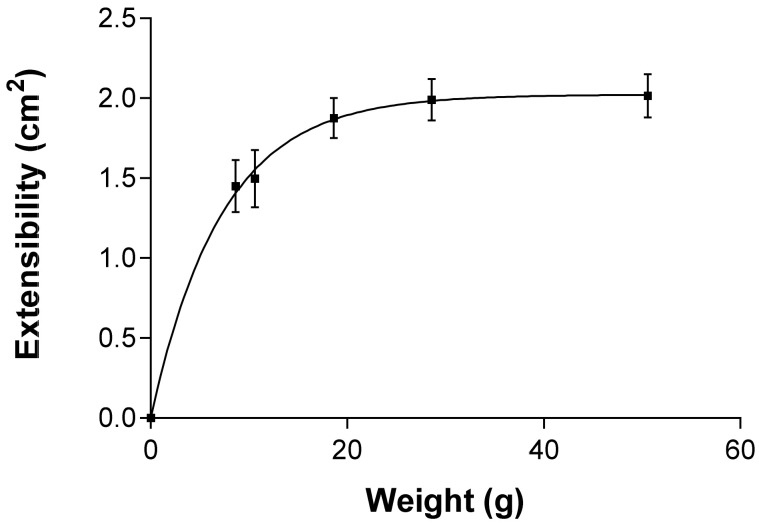
Extensibility profile of PF-NLCs-F127 hydrogel at 25 °C ± 2 °C; 60% ± 5% RH. The data were obtained from the mean of three experiments (*n* = 3).

According to the mathematical modelling, PF-NLCs-F127 hydrogel showed a first order (one-phase exponential association) kinetic profile (Figure 5). The results are expressed as mean ± standard deviation of three replicates (*n* = 3). The value obtained for the maximum extensibility (Ymax) was 2.023 ± 0.088 cm^2^ and the constant (K) was 0.138 ± 0.022 g*^−^*^1^.

#### 2.2.7. Short-Term Stability Studies

**Figure 6:**
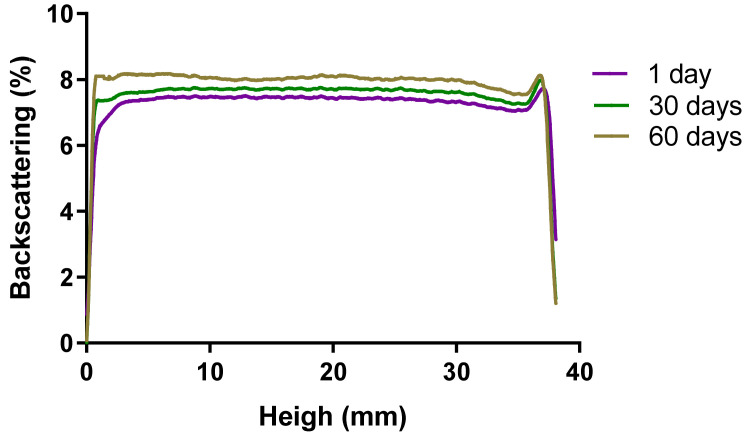
PF-NLCs-F127 hydrogel backscattering profiles obtained by the Turbiscan*^®^*Lab (Formulaction Co., L’Union, France) stored at 25 °C. The left side of the curve corresponds to the bottom of the vial, whereas the right side corresponds to the sample behaviour at the top.

Short-term physical stability was evaluated from the backscattering profiles obtained by the Turbiscan^®^Lab (Formulaction Co., L’Union, France) storing samples at 25 ± 2 °C; 60 ± 5% RH. A backscattering profile of PF-NLCs-F127 hydrogel revealed variations of less than ±10% 60 days after production, indicating that the formulation was stable (Figure 6). No sedimentation, flocculation, or coalescence phenomena were observed.

### 2.3. In Vitro Release Studies

The best-fitting order was chosen according to the coefficient of determination (r^2^). PF released from the hydrogel followed a first order (one-phase exponential association) kinetic model, and the release parameters can be seen in Table 2.

This model presented the smallest value of the AIC (Akaike*’*s information criterion, a discriminatory parameter, which is a measure of the best fit based on the maximum likelihood, comparing several models for a given set of data), and it was also confirmed by the value of the coefficient of determination, r^2^, that was closest to one.

**Figure 7:**
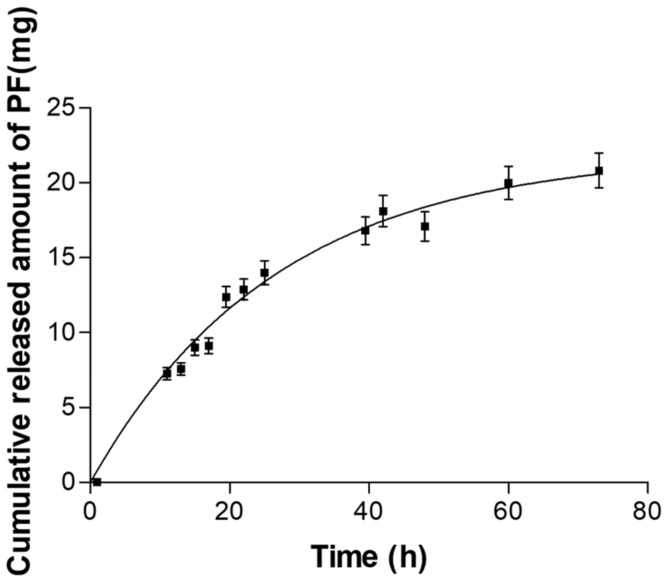
In vitro release profiles of PF from hydrogel. The data were obtained from the mean of three experiments, (*n* = 3) ± standard deviation (SD).

The results showed a release rate constant (K_f_) of 0.037 h*^−^*^1^ and a release half life (t_1/2_) of 18.46 h, which means that the release is slow, and after 18 h of assay, the release rate was further reduced. Despite being a Fick model, it is not very sustained, and therefore, even at 80 h, the value of the asymptote had not been reached, which is around 20 mg. If this value is normalized by the release surface on which it worked, which was 0.64 cm^2^, it is possible to observe that the amount released per surface at 20 h is high enough, and the time in which this was carried out was not a limit on the experiment (Figure 7).

### 2.4. Ex Vivo Permeation Studies

PF-NLCs-F127 hydrogel was tested in the human skin over a period of 36 h. Table 3 exhibits the results obtained from the permeation and prediction parameters of the formulation. The flow (Jss) and the permeability coefficient (Kp) were determined from the cumulative permeated amount of PF (µg) as a function of time. Predicted steady-state plasma concentration (Css) values obtained for the PF-NLCs-F127 hydrogel, considering a hypothetical area of application of 100 cm^2^, were less than the reported therapeutic plasma concentration (4.89 ± 1.29 µg/mL and 10.19 ± 2.43 µg/mL for young and elderly subjects, respectively) [23].

### 2.5. In Vitro Cytotoxicity Studies by MTT Assay

**Figure 8:**
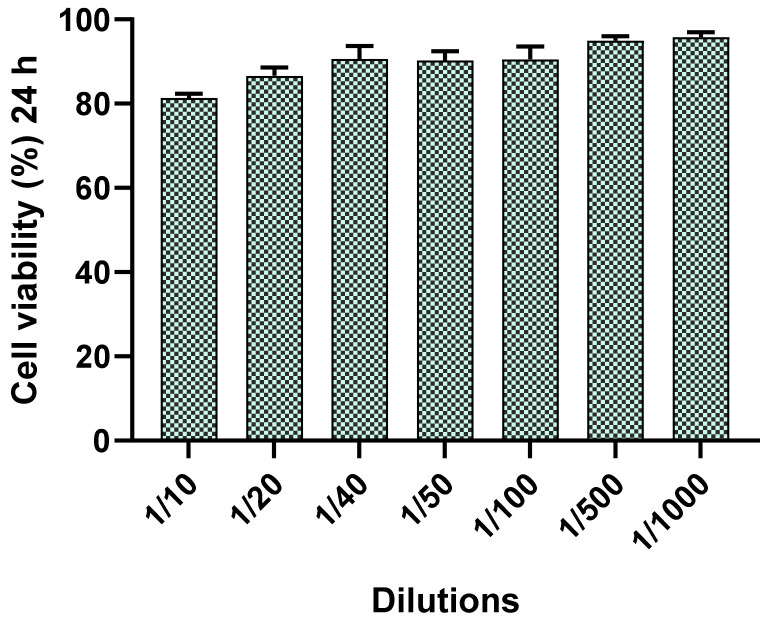
Percentage of cell-viability of HaCaT cell line exposed for 24 h at different concentrations.

The effect of different concentrations of PF-NLCs-F127 hydrogel on human keratinocytes were evaluated using 3-(4,5-dimethylthiazol-2-yl)-2,5-diphenyltetrazolium bromide tetrazolium (MTT), cytotoxicity assay [24]. After 24 h of incubation, it was observed that the assayed dilutions of the formulation (1/10 to 1/1000) did not affect the cell viability, which was close to 95% (Figure 8).

### 2.6. In Vivo Tolerance Study by Evaluating Biomechanical Properties of Human Skin

**Figure 9:**
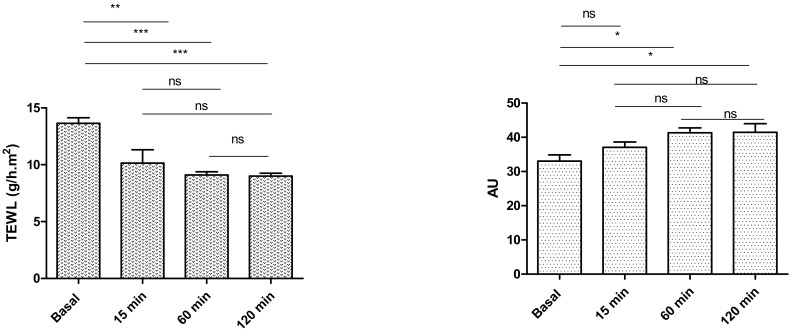
Biomechanical parameters evolution monitored before the application of the formulations and 1 *h* after application. TEWL is expressed as g/h cm^2^ and the SCH as arbitrary units (AU). Significant statistical differences: * *p* < 0.05, ** *p* < 0.01, *** *p* < 0.001, ns = non-significant.

The evolution of biomechanical parameters (transepidermal water loss (TEWL) and stratum corneum hydration (SCH)), before and after the application of the formulation assayed, are shown in Figure 9. Statistically significant decreases in the TEWL values after skin application of PF-NLCs-F127 hydrogel were recorded, which explained an occlusive effect without altering skin integrity. Despite this, the SCH suffered a slight increase. Given that skin capacitance is directly related to skin hydration, these results indicated that the formulations slightly increased the hydration with respect to the normal behaviour of the skin.

In addition, no skin visual irritation was observed after the application of PF-NLCs-F127 hydrogel on the patients’ skins, and thus, this indicates that the hydrogel was well tolerated.

### 2.7. Efficacy Studies: In Vivo Assay and Histological Analysis

**Figure 10:**
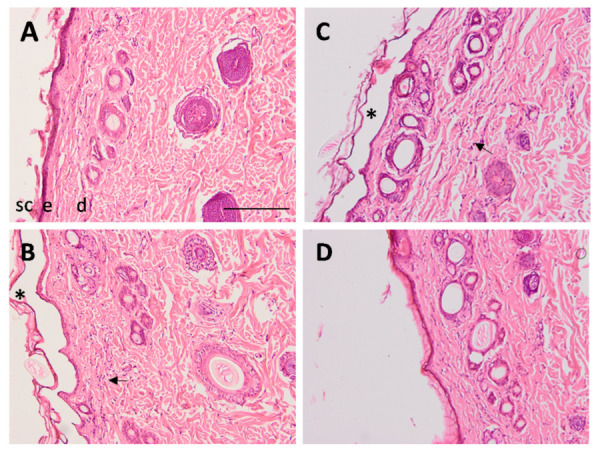
Representative histological sections (original magnification, ×100) of rats’ skin in control conditions (**A**) and after 1 h treatment with xylol; without treatment (**B**), treated with the formulation without active ingredient (**C**), and treated with PF-NLCs-F127 hydrogel (**D**) stained with hematoxylin and eosin. Skin structures: sc, stratum corneum; e, epidermis; d, dermis; * indicates the loss of stratum corneum and the arrow indicates the infiltration of leukocytes. Scale bar = 100 µm.

To investigate the possible anti-inflammatory effect of PF-NLCs-F127 hydrogel, rats’ skin exposed to xylol was used as a model of inflammation. As shown in Figure 10A, rats’ skin in control conditions consisted of an epidermis with a contiguous stratum corneum, a normal epidermis followed by unaltered dermis with dermal appendages, including sebaceous glands and hair follicles.

The architecture of the xylol-treated skin alone (Figure 10B) and with blank hydrogel (Figure 10C) showed signs of irritation with the presence of leukocyte infiltration and the loss of stratum corneum. The administration of PF-NLCs-F127 hydrogel (Figure 10D) prevented the loss of stratum corneum and reduced the presence of infiltrate.

## 3. Discussion

In this work, a topical Pluronic^®^ F127 hydrogel loaded with PF-NLCs prepared by hot homogenization technique was successfully studied. This formulation was designed with the aim of modulating the drug release and promoting its retention in human skin in order to offer an alternative treatment for acute and chronic inflammatory skin diseases. PF-NLCs were previously characterized, which showed a small size of around 248.40 nm, low polydispersity index (0.22), high encapsulation efficiency (99.68%), and good stability (zeta potential of −10.70 mV), which indicates favourable properties for dermal application (Table 1) [21]. Nanosystems are a promising strategy to improve drug penetration throughout the stratum corneum (SC). These nanocarriers facilitate drug delivery and provide controlled release by creating depots on the skin. Specifically, several studies have shown that NLCs enhance drug penetration up to the epidermis, reducing systemic absorption, and providing chemical stability of compounds sensitive to oxidation and hydrolysis [25,26].

PF-NLCs were incorporated into a hydrogel containing Pluronic^®^ F127 (18%), which is a non-ionic triblock copolymer consisting of 70% (*w/w*) PEO units and 30% (*w/w*) PPO units. Pluronic^®^ F127 was used for hydrogel formulation based on its low toxicity, high solubilizing potential and high biocompatibility [27]. Moreover, it is an excipient approved by the United States Food and Drug Administration (FDA) for different types of dermal products and represents an attractive component due to its thermo-reversible properties [28]. The PF-NLCs-F127 hydrogel showed a whitish-translucent colour, homogeneous appearance, and improved consistency with respect to PF-NLCs due to the incorporation of Pluronic F127, facilitating its application while increasing its residence time in the treated skin area. The pH value of this formulation was 6.5, which is suitable for skin administration and suggests a non-irritating effect. Regarding the morphological structure (Figure 2), SEM micrographs of PF-NLCs-F127 hydrogel revealed the typical porous structure that characterizes Pluronic gels [29]. Moreover, PF-NLCs of nanometric size and spherical shape dispersed inside the hydrogel without showing aggregation (none was observed). These characteristics offer advantages in the application of topical formulations, since the small size of the nanoparticles provides a large surface area and uniform distribution on the treated skin area [30].

Pluronic^®^ F127 gels are characterized by their thermo-reversible properties that are manifested by a change from a solution of low viscosity to a semisolid gel at concentrations of this polymer >18% as temperature increases [17]. In this study, PF-NLCs-F127 hydrogel that was liquid at room temperature, showed a gelling temperature of 32.5 °C, close to human cutaneous temperature, which could facilitate its application while increasing its residence time in the treated skin area and during which it ensured its pharmacological efficacy.

In the degradation studies (Figure 3B), PF-NLCs-F127 hydrogel showed values of 1 h for weight loss corresponding to the gradual diffusion of the components of the hydrogel into the medium, indicating that the formulation could be successfully degraded when immersed in the biological medium. The mechanical properties of the gel are also affected by the incorporation of a drug [31]. The high porosity 95.76 ± 0.1%, as well as the hydrophilic behaviour of the fibres, is ideal from a drug delivery point of view, as with these characteristics it could permit the incorporation of solvent, and it explains the high and fast swelling observed, which reached a value of 100% after 7 min. In addition, it could predict a fast diffusion of the drug during drug release experiments.

The rheological studies revealed a pseudoplastic behaviour of PF-NLCs-F127 hydrogel that is frequent in this type of formulation (Figure 4). This feature facilitates skin application since the viscosity of the formulation decreases with friction and returns to the initial state when the friction stops, all of which favours its extensibility capacity and its residence time in the treated skin area, respectively [32,33]. These results were consistent with the spreadability evaluation that showed a maximum extensibility of around 2 cm^2^ (Figure 5), which suggests that PF-NLCs-F127 hydrogel is easy to apply.

The short-term stability of the PF-NLCs-F127 hydrogel (Figure 6) did not detect signs of destabilization over 60 days at 25 °C since the BS profile revealed variations of less than ±10%, indicating that the formulation was stable [34,35].

The in vitro release study revealed information about the capacity of the formulation to release the drug incorporated and it was used to predict in vivo behaviour. In this study, PF-NLCs-F127 hydrogel was able to release an amount of 20 mg of PF, which represents 93.46% of the drug placed in the donor compartment (Figure 7). It has been possible to determine that the release profile followed a first-order kinetic model with a constant slow release (K_f_) of 0.037 h^−1^ and a release t_1/2_ of 18.46 h. (Table 2). This fact allows the prolonged release of the drug, and therefore the improvement of the biopharmaceutical profile of PF. First order means that the PF is delivered at a rate proportional to the concentration gradient driving the transfer of the drug movement, based on the first Fick’s law, where the released amounts are directly proportional to the amounts remaining in the hydrogel.

The results obtained from the ex vivo permeation studies revealed that PF permeated through the human skin in very small amounts, which is indicative that most of the drug would exert its anti-inflammatory activity on the skin locally. In addition, a greater amount of drug retained in the skin (Q_ret_) was observed for PF-NLCs-F127 hydrogel (83.33 µg/g/cm^2^) with respect to PF-NLCs (24.29 µg/g/cm^2^), confirming that the incorporation of Pluronic^®^ F-127 constitutes a valuable strategy to improve the bioavailability of the drug. These findings can be attributed to the properties of Pluronic^®^ F127. The latter has been reported as an excipient that can enhance the diffusion ability of a drug thanks to its amphiphilic structure, which allows it to act as a surfactant and interact with biological membranes. Finally, the predicted steady-state plasma concentration values (C_ss_) obtained for PF-NLCs-F127 hydrogel were 84.39 × $10^{-3}$ and 158.92 × $10^{-3}$µg/mL for young and elderly subjects, respectively (Table 3); these are much lower than the therapeutic plasmatic concentration (4.89 ± 1.29 µg/mL and 10.19 ± 2.43 µg/mL for young and elderly subjects, respectively) [22]. These results ensure that the application of PF-NLCs-F127 via dermal will not induce a systemic effect of the drug, but the anti-inflammatory effect and the PF analgesic effect will be local.

The tolerance of PF-NLCs-F127 hydrogel was evaluated by in vitro and in vivo experiments. In vitro cytotoxicity results (Figure 8) indicated that PF-NLCs-F127 hydrogel did not trigger a relevant toxic effect on cells, which suggests a high biocompatibility with the human keratinocytes cell line (HaCaT). These findings were supported by evaluation of biomechanical skin properties (TEWL and SCH) in ten volunteers after the application of the PF-NLCs-F127 hydrogel (Figure 9). TEWL values were significantly reduced in all cases, while SCH was slightly increased. These results suggest that this formulation does not cause destabilization or damage to the skin surface and acts as an occlusive moisturizer that physically prevents transepidermal water loss and thus increases the skin hydration, possibly due to the lipid composition of the nanocarriers and the chemical nature of Pluronic^®^ F127 [21,36].

Finally, the anti-inflammatory potential of PF-NLCs-F127 hydrogel was evaluated using an acute inflammation model induced by xylol in rats. This model is widely used as an effective tool for efficacy studies with a good predictive value in the screening of anti-inflammatory products [37]. The topical application of xylol caused histopathological changes, including tissue lesions, mainly the loss of stratum corneum and prominent leukocyte infiltrate as a result of the acute inflammatory response. Conversely, topical treatment with PF-NLCs-F127 hydrogel notably enhanced the histological characteristics of the skin and reduced the leukocyte infiltrate at the area of inflammation.

## 4. Materials and Methods

### 4.1. Materials

PF (CAS 52549-17-4) was kindly supplied by Alcon Cusi (Barcelona, Spain). Polyethylene glycol sorbitan monooleate (Tween^®^ 80), castor oil (*Ricinus communis* L.), Phosphate buffer saline (PBS) pH 7.4 (tablets), and Pluronic^®^ F127 were purchased from Sigma-Aldrich (Madrid, Spain). LAS (PEG-8 Caprylic/Capric Glycerides) and Precirol^®^ ATO 5 (glycerol mono, di, and tripalmitostearate) were given by Gattefosse (Saint-Priest, France). All other chemicals and reagents used in the study were of analytical or high-performance liquid chromatography (HPLC)-grade and provided by Fisher Scientific (Leicestershire, UK). Reagents for histological procedures were purchased from Thermo Fisher Scientific (Barcelona, Spain). MTT assay for cytotoxicity studies was provided by Sigma-Aldrich Chemical Co., (St. Louis, MO, USA) and the Milli-Q water used in all the experiments was obtained from a Milli-Q Plus System (Darmstadt, Germany).

### 4.2. Preparation of NLCs

The NLCs were obtained by a high-pressure homogenization method and optimized as described previously [21]. Briefly, the lipid phase (5 wt % with regards to the total amount of formulation) containing LAS: Castor oil (75/25) as liquid lipid (LL) and Precirol^®^ ATO 5 as solid lipid (SL) and 1.5 wt% of PF, were melted in a water bath at 85 °C to obtain a homogeneous lipid solution. An aqueous solution with Tween^®^ 80, heated at the same temperature, was added to the hot lipid phase, and a pre-emulsion was generated using an Ultra-Turrax T25 (IKA, Staufen, Germany) at 8000 rpm for 45 s, which was passed through a high-pressure homogenizer (Homogeniser FPG 12800, Stansted, UK). The production conditions were three homogenization cycles, at 800 bar and 85 °C. Afterward the nanoemulsion obtained was cooled down to room temperature, recrystallizing the lipid, and forming the NLCs. All the characterizations regarding size, PI, ZP, and %EE were as described before [21].

### 4.3. Preparation of Pluronic^®^ F127 Hydrogel Loaded with PF-NLCs

For the hydrogel preparation, Pluronic^®^ F127 was slowly added to previously cooled Milli-Q water until a final concentration of 18% of Pluronic^®^ F127 was obtained, since the latter is the minimum concentration required to obtain an aqueous solution at 4–5 °C and which turns into a gel at ~32 °C. This mix was kept under magnetic stirring at a constant speed until the complete solubilization of the polymer had been achieved. The Pluronic^®^ F127 hydrogel was kept refrigerated for 24 h. Finally, 5 g of the NLCs was added to 2 g of the hydrogel under magnetic stirring until complete homogenization had been achieved and a final formulation of 10.7 mg PF/g gel was obtained in order to keep an effective drug concentration.

### 4.4. Physicochemical Characterization of PF-NLCs-F127 Hydrogel

#### 4.4.1. pH and Morphological Characterization

The pH value of the formulation was determined using a calibrated digital pH meter GLP 22 (Crison Instruments, Barcelona, Spain).

The Pluronic^®^ F127 hydrogel containing the PF-NLCs was dried beforehand using a vacuum system for 8 h, and after that it was analysed by scanning electron microscopy (SEM) [38]. A small quantity of PF-NLCs-F127 hydrogel was coated with carbon as a conductor agent, and the structure was examined using a JEOL J-7100F (JEOL USA, Peabody, MA, USA).

#### 4.4.2. Gelling Temperature

The hydrogel was introduced into a falcon tube, and it was placed in a water bath, gradually increasing the temperature from 22 °C until gelation was achieved. The temperature of the hydrogel was recorded using a thermometer. The gelation temperature was determined, which is when the formulation changed its phase from liquid to gel.

#### 4.4.3. Swelling, Degradation, and Porosity Studies

The carry out the swelling study, 0.5 g of dried PF-NLCs-F127 hydrogel was used. It was accomplished by a gravimetric method and expressed as the swelling ratio (SR). For the degradation studies, 0.5 g of fresh PF-NLCs-F127 hydrogel was used and the results were expressed as a percentage of weight loss (WL).

In both experiments, the sample was incubated in PBS (pH = 5.5) at 32 °C for 30 min. Samples were removed and weighed after blotting the surface water at predetermined time intervals. The data were obtained from the mean of three experiments, *n* = 3.

The porosity percentage is based on the placing of the PF-NLCs-F127 hydrogel previously dried in absolute ethanol for 20 min and weighing it every 2 min at which time the excess of ethanol is blotted.

The SR ratio was calculated using the following equation and expressed by kinetic modelling:

(1) $$SR=\frac{Ws\;–\;Wd}{Wd}$$

where *Ws* is the weight of the swollen PF-NLCs-F127 at 5 min intervals, and *Wd* is the weight of dried hydrogel.

The degradation (*WL*, %) test was calculated in accordance with the equation and expressed by kinetic modelling.

(2) $$WL\;\left(\%\right)=\frac{Wi\;–\;Wd}{Wi}\times \;100$$

where *Wi* is the initial weight of PF-NLCs-F127, and *Wd* is the weight of the hydrogel at different times.

The porosity percentage (*p*) was calculated by the equation:

(3) $$P\;\left(\%\right)=\frac{W2-W1}{ð\times V}\times 100$$

where *W*1 stands for the weight of the dried PF-NLCs-F127, *W*2 is the weight of the PF-NLCs-F127 after each immersion every 2 min, ð is the density of ethanol, and *V* is the volume of the PF-NLCs-F127.

#### 4.4.4. Rheological Behaviour

Rheological properties studies of PF-NLCs-F127 hydrogel were performed using a rheometer HAAKE Rheostress (Thermo Fisher Scientific, Karlsruhe, Germany) equipped with a cone-plate geometry set-up with a fixed lower plate and an upper cone (Haake C60/2Ti, 6 cm diameter). The temperatures sweep test was performed to determine the transition sol-gel in situ of hydrogel.

For rotational testing, viscosity curves, and flow curves were recorded for 3 min during the ramp-up period from 0 to 50 s^−1^, 1 min at 50 s^−1^ (constant share rate period) and finally 3 min during the ramp-down period from 50 to 0 s^−1^. The data were obtained from the mean of three experiments, (*n* = 3).

To determine the type of hydrogel flow, the data from the 1 (ascending) run and the 3 (descending) runs of the flow curves were fitted to different mathematical models (Newton, Bingham, Ostwald-de-Waele, Herchel-Buclkley Casson and Cross), the best adjusted is the one that gives the highest value of correlation coefficient (r). On the other hand, the average value of viscosity was determined from the data of Section 3 of the viscosity curves.

For oscillatory testing, the stress sweep test (increase of the oscillation angle) was carried out at a constant frequency of 1 Hz within a stress range of 0.1 to 500 Pa in order to determine the zone of linear viscoelasticity, at which points it reaches critical stress.

The subsequent frequency sweep test was performed between 0.01 and 10 Hz at a constant effort (selected within the linear viscoelasticity zone provided by the study above), in order to determine the variations of the elastic modulus (G′) and the modulus viscous (G″), as well as the complex viscosity (η *) at 32 °C, as a function of the frequency.

#### 4.4.5. Extensibility (Spreadability)

This assay was carried out at 25 ± 2 °C and a relative humidity (RH) of 60% ± 5%. An amount of 0.05 g of hydrogel was placed within a 10 cm diameter circle pre-marked on a glass plate, over which another glass plate was placed, as centred as much as was possible. Increasing standard weight pieces (5, 10, 15, 25, and 50 g) were added by resting them on the upper glass plate for 60 s. The increase in the diameter, due to the hydrogel spreading, was noted. The formulation was analysed in accordance with the best kinetic model. The data were obtained from the mean of three experiments, (*n* = 3).

#### 4.4.6. Stability Studies

Short-time physical stability for the PF-NLCs-F127 hydrogel was assessed at 25 ± 2 °C and 60 ± 5% RH by multiple light scattering analysis of backscattering (BS) profiles of a pulsed near-infrared light source at wavelength λ = 880 nm using the Turbiscan^®^ Lab (Formulaction Co., L’Union, France). Data were recorded at 1 day, 30 days and 60 days [39].

### 4.5. In vitro Release Studies of PF-NLCs-F127 Hydrogel

The PF-NLCs-F127 hydrogel release assay was carried out in amber glass Franz diffusion cells (FDC 400, Crown Glass, NJ, USA) using dialysis membranes (MWCO 12,000–14,000 Da Dialysis Tubing Visking, Medicell International Ltd., London, UK), previously hydrated in methanol:water (7:3; *v/v*) for 24 h. The PBS, (pH 7.4) kept at 32 ± 0.5 °C, was used as receptor medium that was continuously stirred at 700 rpm assuring sink conditions.

An amount of 2 g of formulation was placed in the donor compartment in direct contact with the membrane (0.64 cm^2^), then 300 µL of the sample was collected with a syringe from the receptor compartment at predefined time intervals, and the volume withdrawn was replaced by an equivalent volume of fresh PBS (pH 7.4). The PF released was analysed by HPLC, using the method described previously [6]. The data obtained were adjusted to different kinetic models (zero order, first order, Korsmeyer-Peppas and Weibull functions) by nonlinear least-squares regression with appropriate statistical software (WinNonLinR version 3.3, Pharsight Co., Sunnyvale, CA, USA) [6,21]. The results were obtained from the mean of three experiments, (*n* = 3).

### 4.6. Ex Vivo Skin Permeation Study

Human skin was obtained from abdominoplasties practiced on healthy women (Barcelona SCIAS Hospital, Barcelona, Spain). The Bioethics Committee of the Barcelona-SCIAS Hospital approved the Study protocol (Nº002; dated 17 January 2020), and the women provided written informed consent forms. The skin was stored at −20 °C until the experiments were carried out. The skin was dermatomed (GA630, Aesculap, Tuttlingen, Germany) at 0.4 mm-thick and placed between the receptor and donor compartments [40].

Amber vertical glass Franz diffusion cells (FDC 400; Crown Glass, Somerville, NJ, USA) were used with a diffusion area of 0.64 cm^2^. PBS (pH 7.4) was used as a receptor medium. An amount of 0.1 g of PF-NLCs-F127 hydrogel was added in the donor compartment in direct contact with the skin. Then, 300 µL of samples were withdrawn from the receptor compartment at selected times and replaced with receptor medium. At the end of the experiment, the skin was cleaned using a 0.05% solution of sodium lauryl sulphate and distilled water. The diffusional area of the skin in direct contact with the formulation was isolated, weighed, and treated with methanol:water (50:50, *v*:*v*) under sonication in an ultrasound bath for 15 min in order to determine the amount of PF retained in the skin (*Q_ret_*). The steady-state flux across the skin (*J_ss_*, µg/h/cm^2^) and the transdermal permeability coefficient (*K_p_*, cm/s) were obtained by applying the following equations [41]:

(4) $$J_{ss}=\frac{Qt}{A}\cdot t\;$$

(5) $$K_{p}=\frac{J_{ss}}{C_{0}}$$

where *Qt* is the quantity of drug permeated across the skin and thus detected in the receptor compartment (μg), *A* is the active cross-sectional area accessible for diffusion (cm^2^), *t* is the time of exposure (h), *J_ss_* is the steady-state flux across the skin and *C*_0_ is the initial drug concentration in the donor compartment.

Considering the pharmacokinetic parameters of the PF for the young and elderly subjects, the predicted *C_ss_* (theoretical steady-state plasma concentration) of the drug that would penetrate the skin after topical application was obtained using the following equation:

(6) $$C_{ss}=\;J_{ss}\;\cdot \;\frac{A}{CLp}$$

where *J_ss_* is the flux, *A* is the hypothetical area of application (in this case, 100 cm^2^), and *CL_p_* is the plasmatic clearance (1146.60 cm^3^/h and 609.00 cm^3^/h, for both the young and elderly subjects, respectively) [41].

### 4.7. In Vitro Cytotoxicity Studies with the MTT Assay

MTT cytotoxicity assay was carried out employing human keratinocytes cell line, HaCaT. Cells were adjusted at 2 × 10^5^ cell/mL, seeded in a 96-well plate for 24 h in Dulbecco’s Modified Eagle’s Medium (DMEM) with high glucose content buffered with 25 mM HEPES, supplemented with 1% non-essential amino acids, 100 U/mL penicillin, 100 g/mL streptomycin, and 10% heat inactivated foetal bovine serum (FBS). Cells were incubated for 24 h with various dilutions of PF-NLCs-F127 (from 1/10 to 1/1000). HaCaT cells were washed with 1% sterile PBS and incubated for 2 h at 37 °C with MTT solution (2.5 mg/mL). The medium was withdrawn and 0.1 mL of solubilization reagent (99% Dimethyl sulfoxide (DMSO)) was added to lyse the cells and dissolve the purple MTT crystals. The medium was then measured at 570 nm in a microplate photometer Varioskan TM LUX (Thermo Scientific, Waltham, MA, USA). The results obtained using the following equation were expressed as a percentage of cell survival, relative to the negative control (untreated HaCaT cells; 100% viability) [23]:

(7) $$\%\;Cell\;viability=\left(Abs\;sample\right)/\left(Abs\;control\right)\;\times \;100$$

### 4.8. In Vivo Tolerance Study by Evaluating Biomechanical Properties of Human Skin

Human in vivo assay was approved by the Ethics Committee of the University of Barcelona (IRB00003099), and they followed the recommendations of the Declaration of Helsinki [42]. Ten volunteers in the age range of 27–65 years were recruited after medical screening and informed of the nature of the study and of the procedures involved (written informed consent). They were warned not to use skin-care cosmetics on the flexor side of the left forearm for the 12 h before the study, and they were asked to be in the test room (22 ± 2 °C and 40–50% RH) for at least 20 min before the measurements were taken. Circles around 5 cm in diameter were made with a marker and 0.5 g of PF-NLCs-F127 hydrogel were deposited in the middle of the circle by means of a soft circular movement with the thumb to facilitate the distribution of the formulation. A total of 20 circles were performed in a clockwise direction. Measurements were performed before applying the formulation, at 15, 60, and 120 min after application. The measurement of the quantity of water that passed through the epidermal layer of the skin into the surrounding atmosphere by diffusion and evaporation processes was performed by the TEWL assessment using a DermaLab^®^ module (Cortex Technology, Hadsund, Denmark) [42]. To measure the TEWL, the probe, a small hollow cylinder, was held on the skin surface for 2 min. TEWL values (g/h) were reported as the mean of 10 replications ± SD. The measurements of SCH were performed using a Corneometer^®^ 825, which was mounted on a Multi Probe Adapter^®^ MPA5 (Courage & Khazaka Electronics GmbH, Cologne, Germany). The measurement was performed by the capacitance method, which used the relatively high dielectric constant of water compared to those of other substances of the skin. Values (arbitrary units, AU) were reported as the mean of 10 replications ± SD.

### 4.9. In Vivo Assay and Histological Analysis

The anti-inflammatory effect of the formulation was evaluated in adult male Sprague Dawley^®^ rats using a model of inflammation induced by topical application of xylol for 1 h. This assay was carried out following a previously approved protocol by the Animal Experimentation Ethics Committee of the University of Barcelona (Nº: 387/18; dated 26 November 2018). The histological observation of the skin architecture was performed using haematoxylin and eosin staining. The rats’ skin of dorsum was shaved and cleaned with 70% ethanol and then irritation was induced by xylol exposure for 1 h. Then, the formulation both with or without the active ingredient was applied for 1 h. Finally, rats were euthanized, and skin was surgically excised, rinsed with PBS and set overnight in 4% buffered formaldehyde at room temperature. After fixation, all samples were paraffin embedded onto cassettes, sectioned into 5 µm slices, mounted on microscope slides and stained with haematoxylin and eosin, and finally viewed under a microscope (Olympus BX41 and camera Olympus XC50, Olympus, Barcelona, Spain) [43].

## 5. Conclusions

The present study provides evidence that the dermal application of PF-NLCs-F127 hydrogel could constitute an effective system for the controlled release of PF by this route. Moreover, this formulation facilitates the contact of PF with the skin, improving its dermal retention, biopharmaceutical profile, and reducing the associated adverse effects in comparison to NSAIDs.

These promising results encourage further clinical investigation into the results of employing this formulation for local inflammation to treat conditions such as atopic dermatitis, rosacea, vitiligo, psoriasis, eczema, and panniculitis.

## Figures and Tables

**Table 1:** Composition and physicochemical characterization of the selected nanostructured lipid carriers of Pranoprofen (PF-NLCs). The data were obtained from the mean of three experiments (*n* = 3).

Composition of PF-NLCs			Physicochemical Characterization			
cPF (%)	cSL/L (%)	cTW (%)	Z-Ave (nm) ± SD	PI ± SD	ZP (mV) ± SD	EE (%) ± SD
1.50	50.00	2.50	248.40 ± 6.34	0.22 ± 0.04	−10.70 ± 0.44	99.68 ± 0.17

**Table 2:** In vitro release profile. Average parameters obtained after fitting the drug release data to different equations of the kinetic model. (The data were obtained from the mean of three experiments, *n* = 3).

Order Equation		Parameters	Unit Value (Mean ± SD)	
Zero order	Q_t_ = K_0_ t + Q_∞_	K_0_	mg/h	0.26 ± 0.02
		Q_∞_	mg	4.98 ± 0.62
		r^2^	-	0.8395
		AIC	-	152.98
First order	Q_t_ = Q_∞_(1 − e^−kf·t^)	K_f_	h^−1^	0.037 ± 0.004
		Q_∞_	mg	22.01 ± 1.05
		r^2^	-	0.9227
		t ½	h	18.46
		AIC	-	132.53
Korsmeyer-Peppas	Qt = K_k_ t *^n^*	K_K_	h^−n^	2.379 ± 0.281
		n	-	0.52 ± 0.03
		r^2^	-	0.9182
		AIC	-	134.14
Weibull	Q_t_ = Q_∞_(1 − e^−(t/td)β^)	t_d_	h	39.85 ± 19.06
		β	-	0.79 ± 0.13
		Q_∞_	mg	26.36 ± 5.49
		r^2^	-	0.9262
		AIC	-	196.84

Qt = cumulative percentage of drug release at time t; Q_∞_ = maximum amount of dissolved drug; K_0_,K_f_,K_k_ = release rate constants; t = time in hours; β = shape parameter; t_d_ = dissolution time; r^2^ = determination coefficient; AIC = Akaike’s information criterion; SD = standard deviation.

**Table 3:** Permeation parameters obtained after ex vivo study. Predicted steady-state plasma concentration (C_ss_) of the PF after application of the formulation on 100 cm^2^ of skin. Results are reported as the median, minimum, and maximum range values. The data were obtained from the mean of six experiments (*n* = 6).

J_ss_ (µg/h/cm^2^)	K_p_ (cm/h) 10^5^	Q_ret_ (µg/g/cm^2^)	Q_24h_ (µg)	Young Subject $\mathrm{Css}\;(\text{µ}g/\mathrm{mL})\;\times \;10^{3}$	Elderly Subject $\mathrm{Css}\;(\text{µ}g/\mathrm{mL})\;\times \;10^{3}$
1.134 (1.129–1.140)	59.5 (59.3–59.8)	83.33 (82.00–99.50)	27.95 (23.10–28.02)	84.39 (76.49–90.83)	158.92 (144.03–171.04)

## Data Availability

Data is contained within the article.
